# Supplementation with Vitamin B6 Reduces Side Effects in Cambodian Women Using Oral Contraception

**DOI:** 10.3390/nu6093353

**Published:** 2014-08-26

**Authors:** Chivorn Var, Sheryl Keller, Rathavy Tung, Dylan Freeland, Alessandra N. Bazzano

**Affiliations:** 1 Reproductive Health Association of Cambodia, No. 14, St. 335, Phnom Penh 12151, Cambodia; E-Mails: chivorn@rhac.org.kh (C.V.); sheryl@ksc.th.com (S.K.); 2 National Center for Maternal Child Health, Ministry of Health, Royal Government of Cambodia, No. 31A, St.47, Phnom Penh 12151, Cambodia; E-Mail: rathavy@online.com.kh; 3 Department of Global Community Health and Behavioral Sciences, School of Public Health and Tropical Medicine, Tulane University, 1440 Canal St, New Orleans, LA 70112, USA; E-Mail: dfreel@gmail.com

**Keywords:** pyridoxine, Vitamin B6, combined oral contraceptive, side effects, family planning

## Abstract

Hormonal contraceptives may produce side effects that deter women from their use as a method of family planning. In nutritionally vulnerable populations these effects may be more pronounced due to micronutrient deficiencies and health status. Previous studies have been unable to resolve whether micronutrient supplementation may reduce such side effects. Aim: In a longitudinal study, 1011 women obtaining oral contraception through the public health system in rural Cambodia were allocated to either intervention or control groups, receiving either daily Vitamin B6 supplement or care as usual (without placebo). Results: The intervention participants (*n* = 577) reported fewer side effects in three categories: nausea/no appetite, headache, and depression compared with control group participants (*n* = 434). Conclusion: Women taking Vitamin B6 supplement were less likely to report side effects in a nutritionally vulnerable population. Underlying nutrition status should be considered by clinicians and reproductive health policy makers in the context of providing contraceptive services. Further investigation into micronutrient supplementation, particularly with B6, in reproductive-aged women using hormonal contraception should be conducted in other settings to determine the potential for widespread adoption.

## 1. Introduction

Modern family planning services are clearly linked with improved population level health outcomes, including reductions in maternal and neonatal mortality and improvements in nutritional status [1,2]. In Cambodia, modern methods of family planning have yet to be widely adopted by the population. Although rising among married women from 19% to 35% since 2000, the prevalence of modern method contraceptive use is not on track to meet the country’s Millennium Development Goal of 60% [3,4]. Combined oral contraceptives (COCs) are the most popular of modern methods with 15% coverage. Ease of use, accessibility, and cost have been suggested as reasons for their popularity; however, there are deterrents that prevent further increases in the adoption of oral contraceptives.

Physical side effects appear to play an important role as barriers to oral contraception in Cambodia and other Asian countries. National level data collected by the Ministry of Health identified side effects as a dominant issue in adoption and continuation of family planning. A 2005 survey of perceptions of available contraceptive methods among Cambodian women with unmet family planning needs found that 70% of those who discontinued use of oral contraceptives did so because of side effects (excluding those who stopped use to get pregnant) [5]. There is little documented evidence about the magnitude or severity of these deterring side effects, and they are likely to be different among populations in higher and lower income countries [6].

Vitamin B6 has been widely used to manage nausea and vomiting during pregnancy [7,8], but its utility in reducing contraceptive side effects has not been firmly established. Studies of women from the UK, Brazil, Sudan, and Mexico offer mixed results about how B6 may impact side effects [9,10,11]. These studies, though valid with respect to each population of interest, may not be applicable in Cambodia and there has been a dearth of recent research in this area.

Based on the need for country-specific information about side effects from COC use and the success of a small unpublished pilot study [12], which found B6 significantly reduced reported side effects, the present study sought to determine if B6 supplementation decreased the incidence and severity of oral contraceptive side effects in Cambodian women and, if so, whether it resulted in an improved continuation rate. To our knowledge this is the first study to specifically investigate Vitamin B6 monotherapy for treatment of contraceptive side effects in Southeast Asia.

## 2. Methodology

A longitudinal study was carried out between April 2009 and October 2010 in Kong Pisey Operational District (OD) of Kampong Speu Province, a rural area located in south-central Cambodia. Several criteria contributed to choice of the district, which is representative of rural Cambodian operational districts as whole. These included: limited access to public facility health providers, high use of the private sector for health care contraceptive needs, and an ethnic Khmer population that is predominately Buddhist and employed in subsistence agriculture.

All Health Centers (HCs) serving the OD were allocated, on the basis of balanced distribution, to control or intervention group. The sampling frame consisted of 246,812 women receiving care from 19 Health Centers. All new oral contraceptive patients served through the public sector from 1 May 2009 onward were asked to participate provided they did not have an excluded illness and no oral contraceptives were taken in the past six months. The total catchment area populations for the ten control and nine intervention HCs were 123,442 and 123,370, respectively.

In the intervention area, new oral contraceptive users were instructed to take 25 mg pyridoxine hydrochloride daily (tablet produced specifically for the study by a local pharmaceutical manufacturer, Pharma Product Manufacturing, Phnom Penh, Cambodia), with instructions to consume with food at a different time of day than the contraceptive pill. Study participants were informed that the tablets were a vitamin supplement with unknown impact on side effects. Women in the control group received care as usual. No placebo was given to the control group, as the logistics of ensuring separation of B6 and placebo were prohibitive considering size of implementation through government service delivery channels.

No women refused to participate, and a total of 1022 were initially enrolled. All participants received a contraceptive formulation of 30 mcg ethinyl estradiol and 150 mcg levinorgestrel and instructed to take one tablet per day. Exclusion criteria were: pre-existing illness (including HIV/AIDS diagnosis, active TB, thyroid toxicosis, heart disease, or severe hypertension at the first interview (*n* = 7)); pre-existing pregnancy (*n* = 2); Vitamin B supplement provision problems prevented two additional women from participating in the intervention group (*n* = 2). Thus, a total of 1011 women were included in the study: 577 in the intervention group and 434 in the control group. All of these participants provided informed consent, and the study was approved by Cambodia National Ethics Committee for Health Research.

All health centers had trained health volunteers who conducted community-based sales of oral contraceptives and condoms. Community-based distribution was conducted under the supervision of the health centers and was thus considered part of the formal public sector. Oral contraceptives were also available from midwives at the health centers and in public markets.

### 2.1. Data Collection

Demographic data were obtained using a standardized questionnaire administered in face-to-face interviews at the time of initial enrollment into the study. Data were gathered on: age, parity, ownership of assets and housing material, education, history of prior use of oral contraceptives and, if any, reason for previous discontinuation. Anthropometric measures, including body weight and height and BMI, were also collected at baseline. The questionnaires were completed by trained providers and were re-checked at the first follow-up interview by local research assistants.

Women in the study were followed up periodically through August 2009. Follow-up interviews were conducted at intervals that corresponded to the 1st, 3rd and 6th month of pill use for all women, and all women were enrolled in the study for at least six months. (Women who enrolled in the study before December 2009 also received a fourth final interview which corresponded to the 9th–12th month of use depending on when the women enrolled relative to the end of the observation period.) The same questionnaire was used at all follow-up visits and consisted of questions about oral contraceptive use, Vitamin B6 use in the intervention area, nature of any side effects experienced and any measures taken to deal with them, and the reasons for discontinuation if the woman was found to have discontinued use. Specific health conditions were queried and an “Other” category was included for any side effects not listed. To ensure the quality of interview, research supervisors checked the completion of questionnaires and re-interviewed 5%–10% each month.

Socio-economic status (SES) was measured using a composite index that considered two housing-related variables (the composition of the walls and roof of the home) and seven asset-related variables (the possession of car, motorcycle, cell phone, television, DVD player, tape player and radio by anyone in the household). Given the frequency distributions, the natural log of the inverse proportion of women who had that item was used for each of these nine SES variables. Values were summed to yield a score, which was then divided into quintiles.

The outcomes of the present study were side effects of oral contraceptive use in relation to Vitamin B6 supplementation. Primary outcomes were problems reported by more than 10% of the women: nausea (with or without vomiting), dizziness, headache, hot flashes, depression and menstrual changes (including “irregular menstruation” and “menstruation increased or decreased in amount or duration”).

### 2.2. Statistical Analysis

Differences in baseline characteristics between control and intervention groups were assessed through Chi Square tests, as were differences across key background variables. Multivariate regressions were used to assess the relative contribution of various factors to the specific side effects and to key behaviors. Kaplan-Meier models were used to determine continuation rates by month of use. All analyses were conducted using SPSS 15.0 (SPSS Inc., Chicago, IL, USA).

## 3. Results

The baseline characteristics of intervention and control group participants are described in Table 1. The mean age was 29 years and the mean length of education was approximately four years. Women in the intervention group had a mean parity of 2.5 and in the control group the mean was 2.4. The body mass index, on average was 20 kg/m^2^, and 21% of the women were classified with undernutrition as defined by a BMI of less than 18.5 kg/m^2^. All variables were comparable between the two groups with the exclusion of the history of having previous discontinued oral contraceptives use due to side effects. Participants in the intervention group had significantly greater history of ever stopping oral contraceptives due to side effects than those in control group (*p* value 0.01).

**Figure 1:**
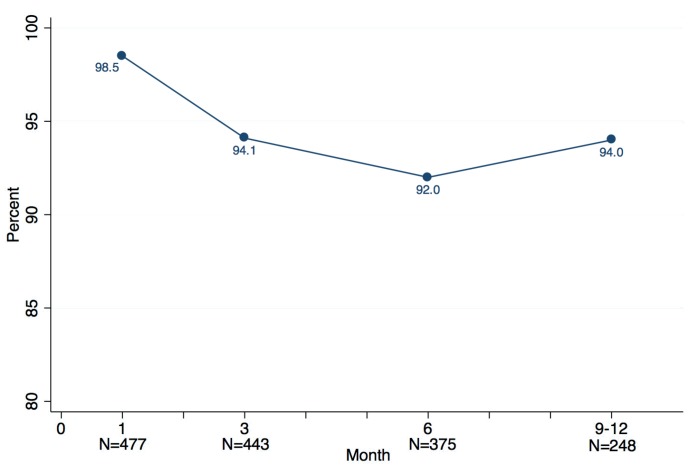
Compliance with Vitamin B6 supplementation.

Figure 1 shows compliance with the Vitamin B6 supplementation in the intervention group. The compliance rate was 98.5% at Month 1, 94.1% at Month 3, 92% at Month 6, and 94% at Month 9 respectively. Although a very small decrease between Months 1 and 3 was observed, the whole curve of compliance remained stable.

**Table 1:** Baseline characteristics by group.

Characteristics	Intervention (*n* = 577)	Control (*n* = 434)
Age, year	29.2	28.3
Length of education, year	3.7	4.2
Body mass index, kg/m^2^	20.5	20.3
Parity	2.5	2.4
Socioeconomic status score	2.8	2.8
History of having previously discontinued oral contraceptives use due to side effects	10.9%	6.2%

Among women who at any time reported they were not taking the supplement (*n* = 89), the most common reason, cited by 50.6%, was an interruption in supply. Most of these occurred in the latter part of the study, *i.e.*, at six months or more of use, at which time temporary logistical problems were encountered in some locations in maintaining the supply chain. The remaining reasons for not taking the Vitamin B6 were fairly evenly divided between forgetting to take the pill, feeling it was unnecessary, and perceived side effects. Only 3.1% of women in the intervention group (*n* = 18) stopped B6 due to perceived side effects, of which weight gain, nausea and gastritis were the most common.

Among intervention and control participants, women with a lower weight or a low Body Mass Index (BMI) reported more side effects than normal weight women at any point in the study. BMI was related to prevalence of nausea among all women in the study, as shown in Table 2. More women reported nausea with a BMI of 18.5–20 than did women with a BMI of 20 or above.

**Table 2:** Prevalence of Nausea by Body Mass Index (BMI).

BMI	No	Yes	Number
Under 18.5	59.9%	40.1%	212
18.5–19.9	56.3%	43.7%	245
20–20.9	68.0%	32.0%	178
21–22.9	67.5%	32.5%	249
23 and over	70.9%	29.1%	127
Total	63.5%	36.5%	1011

Chi Sq = 12.8. *p* = 0.012.

Similarly, more women with low weight reported depression than did normal weight women, as indicated in Table 3 below.

**Table 3:** Prevalence of Depression by Body Weight.

Weight	Any Depression		
	No	Yes	Number
Less than 40 kg	72.2%	27.8%	54
40–44 kg	84.4%	15.6%	250
45–49 kg	82.5%	17.5%	366
50–54 kg	89.0%	11.0%	29
55 kg or more	90.2%	9.8%	122
Total	84.8%	15.2%	1011

Chi Sq = 13.9. *p* = 0.008.

Compared to the women in the control group, participants taking Vitamin B6 had a significantly lower rate of reporting one or more side effect throughout the duration of the study. For specific side effects, there were 48.2% of women in the control group reporting nausea or no appetite, 29.7% reporting headache, and 19.8% reporting depression, compared with 27.4%, 19.8%, and 11.8% in the B6 intervention group, respectively. Reporting of all three side effects was statistically lower in the intervention group with P values ranging from 0.001 to 0.012. Table 4 presents the percentage of all women reporting one or more side effects of any type at 1, 3, and 6 months and Table 5 illustrates any complaint of side effects reported by women in either group.

**Table 4:** Percentage of women reporting 1 or more side effects at 1, 3, 6 months.

Time	Intervention	Control	*p* Value ^1^
1 month	45.2%	68.0%	<0.001
3 month	29.4%	38.7%	<0.001
6 month	27.6%	36.0%	<0.012

^1^*p* values were calculated using chi-square tests.

The most commonly reported side effects among all women in the study were nausea/no appetite, headache and depression. As Table 5 indicates, fewer women in the intervention group reported these common side effects.

**Table 5:** Any complaint of specific side effect by groups.

Side Effect	Intervention	Control	*p* Value ^1^
Nausea/No Appetite	27.4%	48.2%	<0.001
Headache	19.8%	29.7%	<0.001
Depression	11.8%	19.8%	<0.001

^1^*p* values were calculated using chi-square tests.

Despite reducing the prevalence of nausea, headache and depression, B6 supplementation did not have a statistically significant effect on discontinuation due to side effects. The overall one year continuation rate for the study population as a whole was 59%,* i.e.*, 41% discontinued sometime in the first 12 months.

## 4. Discussion

The results of this study provide potentially important information about the use of B6 supplementation for improving the tolerability of contraceptive side effects. To our knowledge, this is the only population-level intervention study on nutritional supplementation and contraception from Cambodia. The information presented on reported side effects, and the possible role of B6 in ameliorating these, is likely to be useful to researchers and policy makers in the region and in other low income, nutritionally vulnerable populations where family planning is important for maternal and child survival [2].

A significant difference between intervention and control in reported side effects was seen particularly for three symptoms: nausea/no appetite, headache, and depression. These three side effects were also most pronounced in women of low weight and BMI. Physical side effects related to contraception may vary by individual and may be related to an individual’s health and nutrition status [13]. Micronutrient deficiencies among Cambodians are endemic and, in particular, B6 and related deficiencies could potentially explain some of the side effects being experienced by women taking oral contraceptives. Undernutrition among women of reproductive age is prevalent in many provinces, and the typical local diet is composed mostly of polished white rice. Anemia among women is 47% in rural areas, where it is estimated that 71% of daily calorie requirement is met by rice [14].

While Vitamin B6 levels have not been assessed nationally in Cambodia, a study of multivitamin supplementation for contraceptive users in Thailand—where the rural diet is somewhat similar—indicated that the population may have been deficient [15]. The authors noted administration of Vitamin B6 to be potentially of benefit, in contrast to multi-vitamin supplementation. Studies of metabolism of B6 in the presence of estrogen are also of interest—links between oral contraceptive use and altered micronutrient levels highlight this relationship [16,17,18,19]. A study in India of oral contraceptive users identified altered tryptophan metabolism and elevated plasma vitamin A levels which were prevented by administering multivitamins containing10 mg Vitamin B6 daily [20].

Results of this study indicated that supplementation reduced the reported side effects of depression and nausea/lack of appetite. Depression has previously been shown in a controlled trial to be responsive to B6 supplementation [9], and nausea has previously been shown in controlled trials to respond to B6 supplementation in the context of pregnancy [7,8]. In contrast to our results, a much smaller trial found no difference in reported side effects among Mexican women assigned to B6 or placebo [21], though nutritional status was not described. Another study, conducted in Canada, reported detection of a disturbance in Vitamin B6 metabolism among young women consuming adequate diets who began a course of low dose oral contraceptive pills for the first time [22].

The major limitations of this study are on the necessity of using self-report via interview questionnaire and the lack of placebo due to logistical constraints. For ethical reasons, it was necessary to inform intervention group participants that the supplement may or may not reduce side effects of contraceptives. In any study relying on self report it is possible that bias may be a factor. In order to minimize measurement error, all the interviewers and other research coordinators were trained and data were checked and rechecked both at collection and prior to analysis. Another potential limitation is lack of data on nutritional status at baseline including Vitamin B6 status or consumption data from relevant local food sources. However, the nutrition situation in Cambodia consistently demonstrates high levels of undernutrition and particularly micronutrient undernutrition among women of reproductive age [23]. Finally, interruption of contraceptive supplies due to logistical issues somewhat limit the interpretation of later results of the study but are an unavoidable consequence of conducting field based research in resource constrained settings.

## 5. Conclusions

Family planning is a key strategy for improving health in low income countries. Women in these countries may be nutritionally vulnerable and may respond to oral contraceptive methods differently than women in higher income settings. The present study assessed effects of oral contraceptive pills in a nutritionally vulnerable population of women in Cambodia and demonstrated that side effects known to impact compliance and deter use of oral contraception may be ameliorated through administration of Vitamin B6. Future research on the dosage, administration and underlying pyridoxine status of contraceptive populations for which B6 supplementation may be considered is warranted, as is research on the possible benefit of multi-vitamin supplementation.
